# Structural covariance, topological organization, and volumetric features of amygdala subnuclei in posttraumatic stress disorder

**DOI:** 10.1016/j.nicl.2024.103619

**Published:** 2024-05-11

**Authors:** Elizabeth M. Haris, Richard A. Bryant, Mayuresh S. Korgaonkar

**Affiliations:** a Brain Dynamics Centre, Westmead Institute for Medical Research, The University of Sydney, Westmead, NSW, Australia; b School of Psychology, University of New South Wales, Sydney, Australia; c Discipline of Psychiatry, Sydney Medical School, Westmead, NSW, Australia; d Department of Radiology, Western Sydney Local Health District, Westmead, NSW, Australia

**Keywords:** Structural covariance, Amygdala subnuclei, Accessory-basal nucleus, PTSD

## Abstract

The amygdala is divided into functional subnuclei which have been challenging to investigate due to functional magnetic resonance imaging (MRI) limitations in mapping small neural structures. Hence their role in the neurobiology of posttraumatic stress disorder (PTSD) remains poorly understood. Examination of covariance of structural MRI measures could be an alternate approach to circumvent this issue. T1-weighted anatomical scans from a 3 T scanner from non-trauma-exposed controls (NEC; n = 71, 75 % female) and PTSD participants (n = 67, 69 % female) were parcellated into 105 brain regions. Pearson’s *r* partial correlations were computed for three and nine bilateral amygdala subnuclei and every other brain region, corrected for age, sex, and total brain volume. Pairwise correlation comparisons were performed to examine subnuclei covariance profiles between-groups. Graph theory was employed to investigate subnuclei network topology. Volumetric measures were compared to investigate structural changes.

We found differences between amygdala subnuclei in covariance with the hippocampus for both groups, and additionally with temporal brain regions for the PTSD group. Network topology demonstrated the importance of the right basal nucleus in facilitating network communication only in PTSD. There were no between-group differences for any of the three structural metrics. These findings are in line with previous work that has failed to find structural differences for amygdala subnuclei between PTSD and controls. However, differences between amygdala subnuclei covariance profiles observed in our study highlight the need to investigate amygdala subnuclei functional connectivity in PTSD using higher field strength fMRI for better spatial resolution.

## Highlights

• Functionally connected brain regions show high covariance in morphological measures.
• Differences between subnuclei covariance profiles in both groups were observed.
• No between-group differences in subnuclei covariance profiles were found.
• This work highlights the merit of examining amygdala subnuclei in humans and PTSD.
• Further work examining functional connectivity using high-field fMRI is necessary.

## 1 Introduction

Posttraumatic stress disorder (PTSD) is a psychiatric disorder that can develop following exposure to a traumatic event. The disorder is typically conceptualized as consisting of impaired fear processing (Careaga et al., 2016), characterized by persistent re-experiencing of traumatic memories of the event, avoidance behaviors, and dysregulated arousal (Sherin and Nemeroff, 2011). The stress response underpinning PTSD comprises neurobiological changes involving brain regions involved in emotion processing (Malikowska-Racia and Salat, 2019). One of the areas that plays a key part in the stress response and is implicated in PTSD, is the amygdala (Malikowska-Racia and Salat, 2019, Hayes et al., 2012, Sartory et al., 2013, Koch et al., 2016). Most studies of the amygdala in PTSD have examined it as a single structure (Hayes et al., 2012, Sartory et al., 2013, Koch et al., 2016, Patel et al., 2012, Wang et al., 2016). This focus on the whole amygdala contrasts with evidence from animal studies that shows differential connectivity patterns of amygdala subnuclei with the rest of the brain and that implicate these subnuclei in differential functions (Janak and Tye, 2015, Mosher et al., 2010, Schmitt et al., 2012). This gap in knowledge is notable considering that models of PTSD posit that subregions of the amygdala are responsible for distinct processes in the acquisition and extinction of fear learning (Ressler et al., 2022). Accordingly, an examination of the connectivity of amygdala subnuclei might contribute to a better understanding of their functional role in PTSD.

Although the human amygdala can currently be delineated into nine subnuclei (Saygin et al., 2017), the most widely accepted division of amygdala subnuclei examined in human neuroimaging studies consists of three larger structures grouped together by similar functions (Amunts et al., 2005). The basolateral amygdala (BLA) is involved in associative learning, particularly the acquisition of conditioned fear, (Myers and Davis, 2007), and shows reciprocal connections with the prefrontal cortex (PFC; 16). The centromedial amygdala (CMA) is involved in the facilitation of behavioral responses, especially fear extinction (Myers and Davis, 2007), and primarily consists of inhibitory neurons connected with brainstem structures (Šimić et al., 2021). The superficial amygdala (SFA) is primarily composed of excitatory neurons, and related to affective, olfactory, and social processing (Šimić et al., 2021). Despite these functional differences, studies investigating amygdala subnuclei functional connectivity in PTSD (Brown et al., 2014, Koch et al., 2016, Leite et al., 2022, Liu et al., 2021, Neumeister et al., 2016, Rabellino et al., 2016, Zhu et al., 2017, Zhu et al., 2018, Nicholson et al., 2015, Nicholson et al., 2017, Nicholson et al., 2016) suffer from a number of limitations, including very small sample sizes (e.g., 10; 19), which might prevent detection of group differences in amygdala activity (Faber and Fonseca, 2014); the use of low-resolution magnetic resonance imaging (MRI) scanners (e.g., 1.5 T scanner; 23, 24), which makes delineating subcortical structures difficult (Sladky et al., 2013); and typical analysis steps, such as smoothing, that further compromise the spatial specificity of the image (Sacchet and Knutson, 2013).

Functional MRI (fMRI) studies of amygdala subnuclei in PTSD (vs. non-trauma-exposed controls [NEC]) show greater functional connectivity between the left BLA and right middle frontal gyrus (Liu et al., 2021, Nicholson et al., 2015) and the right CMA and left middle occipital gyrus (Liu et al., 2021), and lower connectivity of the left SFA and left middle occipital gyrus (Leite et al., 2022). When compared to trauma-exposed controls, individuals with PTSD show greater connectivity between the right BLA and right dorsal anterior cingulate cortex (Brown et al., 2014, Koch et al., 2016) and the default mode network (Brown et al., 2014, Liu et al., 2021), and lower connectivity between the CMA and orbitofrontal cortex/ventromedial PFC (Koch et al., 2016, Zhu et al., 2017, Zhu et al., 2018). One of the major reasons for this paucity of research lies in the limitations of the techniques used. Functional images of subcortical structures that are obtained on 3 T MRI scanners have a lower signal-to-noise ratio due to being smaller and deeper in the brain (Harrison et al., 2021). This complicates the interpretation of connectivity patterns of the amygdala, particularly when exploring individual nuclei. One way of addressing this is to use the finer resolution anatomical scans obtained on 3 T scanners to examine the structural covariance of brain regions.

Just as functionally connected brain regions show temporally correlated blood-oxygen-level-dependent activity at an individual level (Glover, 2011), these brain regions also demonstrate in sync morphological fluctuations at a population level (Alexander-Bloch et al., 2013). These inter-regional associations reflect structural covariance—a phenomenon thought to reflect joint developmental or sustained coactivation processes, and to be akin to both functional and structural connectivity measures (Alexander-Bloch et al., 2013). Structural covariance can be measured using T1-weighted MRI images, which is particularly relevant when examining the connectivity of small structures as T1-weighted images have smaller voxel sizes (often 1 mm^3^ isotropic) and greater spatial resolution than fMRI images (Mulder et al., 2019). Importantly, studies investigating functional connectivity and structural covariance in mental ill-health demonstrate overlapping results and reveal differences in functional networks (Clos et al., 2014, Scheinost et al., 2018), illustrating the complementary nature of both methods in contributing to a richer picture of the functional neurobiology underlying mental ill-health.

Structural covariance has also been used to delineate the topological properties of mental ill health (Wu et al., 2017, Yun et al., 2020, Kim et al., 2020, Sun et al., 2018). Meta-analytic studies in PTSD have shown lower network covariance within the default mode and salience networks (Sun et al., 2022), and higher network centrality for the left fusiform gyrus, left superior temporal gyrus (STG), right inferior temporal gyrus, and occipital areas (Rakesh et al., 2021, Rakesh et al., 2023), results which are consistent with functional connectivity findings (Patel et al., 2012, Akiki et al., 2017). However, these studies often measure cortical thickness, which cannot account for subcortical structures (Carmon et al., 2020). Examining the structural covariance of subcortical regions like the amygdala could further enrich our knowledge of the neural networks underlying PTSD.

The aim of this study was to investigate the structural covariance networks of amygdala subnuclei volume with the rest of the brain in PTSD vs. NEC by using T1-weighted MRI images. As the BLA, CMA, and SFA are commonly investigated, these nuclei were examined first. However, given that the amygdala can be delineated into nine subregions, and that structural scans offer better resolution, we also examined the structural covariance of all nine subnuclei. Based on findings in functional connectivity studies, we expected to see differences between groups in areas involved in cognitive control, visual, reward, salience, and self-referential processing (Leite et al., 2022, Liu et al., 2021, Neumeister et al., 2016, Rabellino et al., 2016, Zhu et al., 2017, Zhu et al., 2018, Nicholson et al., 2015). Additionally, we investigated the topological organization of the brain in PTSD vs. NEC, expecting to find differences between groups for nodes involved in the default mode, salience, and sensory processing networks. Finally, we investigated volumetric differences between amygdala subnuclei to complement these analyses, hypothesizing that there would be no difference between groups as has been found in a recent meta-analysis (Logue et al., 2018).

## 2 Materials and methods

### 2.1 Participants

Data for 71 NEC and 67 participants with PTSD was analyzed for this study. PTSD diagnosis was determined using the Clinical Administered PTSD Scale (CAPS10; (Blake et al., 1995), a structured clinical interview based on DSM-IV criteria. Clinical participants reported PTSD following child abuse (38.8 %), police trauma (20.9 %), assault (19.4 %), domestic violence (7.46 %), road accident (7.46 %), or death of a loved one (5.97 %). Comorbid diagnoses included major depression (67.2 %), social phobia (44.8 %), generalized anxiety disorder (41.8 %), agoraphobia (32.8 %), obsessive–compulsive disorder (16.4 %), and panic disorder (1.49 %). Individuals on a stable dose of psychotropic medication for the past two months were included; those with a history of neurological disorder, psychosis, or substance dependence were excluded.

The study adhered to the Declaration of Helsinki 1975, as revised in 2008, and was approved by the Western Sydney Area Health Service Human Ethics Committee. Written informed consent was obtained from all participants.

### 2.2 Image acquisition and preprocessing

**Fig. 1:**
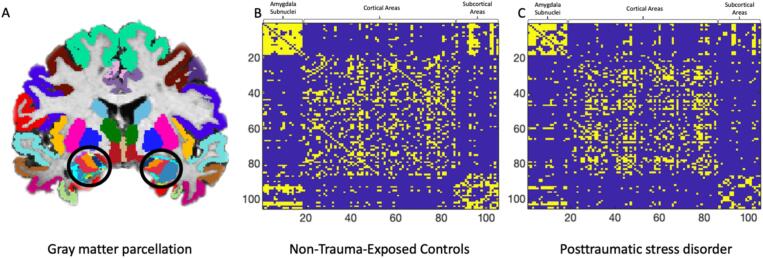
*Parcellation structure generated in FreeSurfer (48) and structural covariance matrices using Graph Analysis Toolbox (GAT;* (Hosseini et al., 2012)*.* Gray matter parcellations for all analyses were obtained from the Desikan-Killiany-Tourville (DKT) atlas. Amygdala subnuclei are circled (A). Binary adjacency matrices when examining structural covariance for nine amygdala subnuclei. Yellow represents connections in non-trauma exposed controls (B) and in posttraumatic stress disorder (C). Maps were calculated by the GAT and thresholded at D_min_ = .19 which designates the minimum network density at which all nodes are fully connected for both graphs. A total of 105 areas were parcellated (a list of all areas can be found in Supplementary Table S1).

Structural T1-weighted MRI images were acquired on a 3 T GE Signa scanner and eight-channel head coil. A total of 180 whole brain slices of 1 mm thickness were acquired for each participant (SPGR, sagittal acquisition, TR = 8.3 ms; TE = 3.2 ms; TI = 500 ms, flip angle = 11 degrees, NEX = 1, ASSET = 1.5, in-plane resolution = 1 × 1 mm^2^, matrix = 256 × 256). Cortical reconstruction and volumetric segmentation of structural MRI images was performed using Freesurfer (version 7.1.0; https://surfer.nmr.mgh.harvard.edu/; 48). For quality control, amygdala subnuclei volumes were checked for major group deviations, and any outliers (outside ±1.5 IQR) were visually inspected for segmentation failures. Additionally, we conducted manual quality checks on a random selection of T1 images from both groups (∼50 %). This involved visual inspection of the cortical, subcortical, and amygdala parcellation/segmentation of gray matter for any major preprocessing issues. The default Freesurfer processing pipeline was used, which includes motion correction, removal of non-brain tissue, segmentation of white and deep gray matter structures, intensity normalization, tessellation of gray matter/white matter boundary, automated topology correction, and surface deformation. The Desikan-Killiany-Tourville (DKT) atlas (Klein and Tourville, 2012), automatic subcortical segmentation in Freesurfer (Fischl, 2012), and volumetric segmentation of amygdala subfields in Freesurfer (Saygin et al., 2017), were used to obtain cortical, subcortical, and subnuclei gray matter volumes, respectively. The DKT atlas was used as it has been found to produce reliable results for structural covariance analyses in sample sizes above 30 participants (Carmon et al., 2020). Gray matter volumes for 63 cortical regions, 24 subcortical regions, and 18 amygdala subnuclei regions of interest were extracted (Supplementary Table S1/Fig. 1). Automated amygdala subfield segmentation in Freesurfer defines nine bilateral subnuclei—anterior-amygdaloid-area, cortico-amygdaloid-transition, accessory-basal, basal, central, cortical, lateral, medial, and paralaminar nuclei. Analyses using three subnuclei combined these nine nuclei into three bilateral traditionally-defined cytoarchitectonic subfields (Amunts et al., 2005): BLA (accessory-basal, basal, lateral, paralaminar), CMA (central and medial), and SFA (anterior-amygdaloid-area, cortico-amygdaloid-transition, cortical). In total, gray matter volumes were obtained for 87 brain regions and nine bilateral amygdalae nuclei (total = 105 regions).

### 2.3 Statistical analysis

#### 2.3.1 Comparison of whole brain structural covariances between amygdala subnuclei and between-groups

Structural covariance was examined using a statistical comparison of the correlations between the gray matter volumes of six amygdala subnuclei (three in each hemisphere) and 87 other brain regions. Pearson’s *r* correlation coefficients were derived for each subnuclei-brain region pair, and were adjusted for age, sex, and total brain volume. Though higher educational attainment has been found to impact brain volume, we did not adjust for education level in our analyses as the effect of education on regional brain size has been reported to be mediated either completely or to a large extent by total brain volume (Kim et al., 2023). To statistically compare the magnitude of correlation coefficients, we used R’s cocor package (Diedenhofen and Musch, 2015, R Core Team, 2020). Cocor performs significance testing between independent (between-group), and dependent (within-group) correlations with non-overlapping (distinct) or overlapping (shared) variables. While the program uses a Fisher’s *z*-transform to ensure near normally distributed variables (Fisher, 1915) and Zou’s confidence intervals to determine both the magnitude and precision of the correlation comparison for independent correlations (Zou, 2007), its advantage lies in performing nine different significance tests comparing dependent overlapping correlations (Diedenhofen and Musch, 2015). Further, for dependent overlapping variables, the package considers the intercorrelation between the dependent variables being compared (i.e., amygdala subnuclei).

Between-group pairwise comparisons of amygdala subnuclei covariances were completed using the cocor.indep.groups function for independent groups. Eighty-seven correlations were calculated for each subnuclei region (e.g., *r*_1_(BLA_1_ x region_1_), *r*_2_(BLA_1_ x region_2_); *r*_3_(BLA_1_ x region_3_), etc…), with the same correlation compared between-groups. Correlations were false discovery rate (FDR) corrected for 87 regions for each subnuclei comparison using R’s p.adjust method (fdr; aka Benjamini, Hochberg, (Benjamini and Hochberg, 1995). Fisher’s *z* statistic (for independent samples) and Zou’s confidence intervals are reported.

Within-group comparisons were also explored to investigate differences in covariance patterns between amygdala subnuclei using the cocor.dep.group.overlap function for dependent groups with overlapping variables. Correlations were FDR-corrected; Dunn’s *z* statistic (for dependent samples) and Zou’s confidence intervals are reported. Dunn’s *z* statistic was selected as it has been shown to have the best statistical properties when compared to most other tests in the cocor package (Hittner et al., 2003).

These analyses were also completed for nine nuclei. Significant *p* values were Bonferroni corrected to account for multiple subnuclei comparisons (*three nuclei*: within-group: *p* ≤ .003 (.05/15); between-group: *p* ≤ .008 (.05/6); *nine nuclei*: within-group: *p* ≤ .0003 (.05/153); between-group: *p* ≤ .003 (.05/18)).

#### 2.3.2 Comparison of amygdala subnuclei topological properties

Graph theory was employed to explore amygdala subregion network topology. Graph theory uses a data-driven approach to quantify and summarize covariance patterns of brain regions into biologically meaningful properties (Alexander-Bloch et al., 2013), and provides several summary measures that describe global and regional network properties (further information in Supplementary Materials).

The graph-analysis toolbox (GAT; 51) was used to compare local topological properties of networks using a structural covariance approach. Whole brain covariance networks were constructed using association matrices of 93 × 93 regions per group, with values representing a Pearson’s correlation coefficient between each pair of regions (Liang et al., 2012). Binary, undirected adjacency matrices were derived for association matrices: values are 1 if greater than a specific threshold and 0 if not. Thresholding of matrices for group comparisons was calculated by the GAT and set using the minimum network density (D_min_) at which both graphs were fully connected, up to the maximum density (D_max_) at which connections become increasingly random (50 %) using steps of 2 % (Diedenhofen and Musch, 2015).

Group topological brain networks were evaluated against 20 null covariance matrices randomly generated and matched to the distributional properties of the observed matrices using the Hirschberger-Qi-Steuer algorithm (Hirschberger et al., 2007). Regional network measures were calculated for nodal degree (the number of connections of a node with the network—to measure nodal importance), betweenness centrality (the number of shortest paths that cross a node—to measure nodes central for network communication), and local clustering (the tendency for nodes that neighbor one another to cluster together to create smaller, complete graphs). Important nodes are considered hubs if their regional degree or betweenness centrality is 2 SD above the group mean (Bassett et al., 2008). Hubs are regions crucial for efficient network communication: nodal degree represents highly connected regions within the network while betweenness centrality represents regions that act as bridges for communication between other nodes within the network (Diedenhofen and Musch, 2015).

Nonparametric permutation tests of 1000 repetitions were conducted and a two-tailed *p*-value calculated. Functional data analysis (FDA) was implemented to control for group comparisons at multiple thresholds (Ramsey and Silverman, 2005). FDR-corrected *p*-values were used to control for multiple comparisons across multiple nodes. A further Bonferroni correction was applied to control for the three regional measures that were examined (*p* ≤ .02).

The above analyses were repeated for nine nuclei.

#### 2.3.3 Comparison of amygdala subnuclei volumes

Subnuclei volumes extracted from Freesurfer were compared between-groups using linear regression models in R (Fox and An, 2019). Six tests were conducted for three bilateral nuclei, and 18 tests for nine bilateral nuclei, with age, sex, and total brain volume included as covariates. Results were Bonferroni corrected for multiple comparisons (three nuclei: *p* ≤ .008; nine nuclei: *p* ≤ .003).

## 3 Results

### 3.1 Sample characteristics

Demographics and summary statistics for both groups can be found in Table 1. Groups did not differ in sex, years of education, or total brain volume, but they did differ on age such that the PTSD sample was slightly older than the NEC group. There were no between-group differences in whole amygdalae volume, but both groups demonstrated higher within-group volume of the right amygdala. Though we didn’t include education level in our main analyses, included in the supplementary materials are the results from a supplementary linear regression model that included the effect of education on subnuclei volumes. For every analysis, total brain volume significantly affected subnuclei volume, but education did not. (Table S2).

**Table 1:** Sample characteristics.

	**NEC** **(71)**	**PTSD** **(67)**	**Stats** **(W/H/***χ*^2^)	***p* value**
**Age (years; Median(IQR))**	35.7 (12.3)	39.4 (11.4)	1872	.03* (NEC < PTSD)
**Sex (F; %)**	53 (75)	46 (69)	0.61	.44
**Education (No/%)**			0.56	.46
Secondary/High School	4 (5.63)	19 (28.4)	−	−
Trade qualification	0 (0.00)	2 (2.99)	−	−
Certificate/Diploma	19 (26.8)	24 (35.8)	−	−
Graduate degree	25 (35.2)	13 (19.4)	−	−
Postgraduate degree	19 (26.8)	4 (5.97)	−	−
Other/Prefer not to answer	1 (1.41)	0 (0.00)	−	−
Not answered	3 (4.23)	5 (7.46)	−	−
**Total Brain Volume** **(mm^3^, Mean(SD))**	1,531,458 (169109)	1,512,241 (134480)	2477	.68
**Left Amygdala Volume** **(mm^3^, Mean(SD))**	1530 (2 0 2)	1504 (2 0 1)	0.77 (1 3 6)	.45
**Right Amygdala Volume** **(mm^3^, Mean(SD))**	1699 (2 0 9)	1670 (2 0 1)	0.80 (1 3 6)	.42
**Within-group Amygdala Volume**				
NEC	−	−	−4.86 (1 4 0)	<.001^**^ (left < right)
PTSD	−	−	−4.78 (1 3 2)	<.001^**^ (left < right)

*Note.* NEC = non-trauma-exposed controls. PTSD = posttraumatic stress disorder. F = female. Statistical tests performed were the nonparametric Mann-Whitney U (W) and Kruskal-Wallis (H) tests, and chi-square test, performed in R. *significant at p < .05. **significant at p < .001.

### 3.2 Analysis of three nuclei

#### 3.2.1 Comparison of whole brain structural covariances between amygdala subnuclei and between-groups

Greater covariance was found between the right BLA (vs. left CMA) and the right amygdala for NEC (*r*_1_ = .81, *r*_2_ = .40; Dunn z = 4.44; *p*_FDR_ < 0.001). In contrast, several differences were found in PTSD, including greater covariance between bilateral BLA (vs. bilateral CMA) and bilateral amygdalae, bilateral temporal gyri, right superior frontal gyrus (SFG), and right middle temporal gyrus (MTG; all *p*’s ≤ .002); and between bilateral SFA (vs. bilateral CMA) and bilateral amygdalae and right hippocampus (all *p*’s ≤ .003; Table 2).

**Table 2:** Significant differences in the covariance of brain regions with three amygdala subnuclei in individuals with posttraumatic stress disorder.

**Amygdala Nucleus 1**	**Amygdala Nucleus 2**	**Brain Region**	***r*1**	***r* 2**	**z statistic**	***p* value**
Left Basolateral	Left Centromedial	Left Amygdala	.77	.37	4.69	.0002
		Right Amygdala	.71	.34	4.13	.002
		Right Superior Frontal Gyrus	.31	–.15	4.02	.002
		Left Superior Temporal Gyrus	.42	–.01	3.81	.002
		Right Superior Temporal Gyrus	.41	–.02	3.79	.002
		Right Middle Temporal Gyrus	.41	–.03	3.83	.002
	Right Centromedial	Left Amygdala	.77	.19	4.93	.00007
Right Basolateral	Left Centromedial	Right Amygdala	.82	.34	4.60	.0004
	Right Centromedial	Right Amygdala	.82	.25	5.26	.00001
Left Superficial	Left Centromedial	Left Amygdala	.75	.37	4.10	.003
		Right Amygdala	.72	.34	3.98	.003
	Right Centromedial	Left Amygdala	.75	.19	4.92	.00008
		Right Amygdala	.72	.25	4.09	.002
	Right Superficial	Left Amygdala	.75	.41	4.24	.002
Right Superficial	Left Centromedial	Right Hippocampus	.64	.04	4.19	.002

Note. *r* = Pearson’s correlation. *r*1 = correlation between amygdala nucleus 1 and brain region. *r*2 = correlation between amygdala nucleus 2 and brain region. Z statistic is based on Dunn’s z. *p* value was FDR-corrected for the number of brain regions compared (1 0 5) and further Bonferroni-corrected for multiple subnuclei comparisons (significant *p***≤**.003).

No group differences survived corrections for multiple comparisons (uncorrected results in Supplementary Table S3).

#### 3.2.2 Comparison of amygdala subnuclei topological properties

Hub FDA designated the right BLA as important for nodal betweenness in PTSD. No subnuclei hubs were found for NEC. Group differences for regional measures did not survive FDR-correction for multiple comparisons (uncorrected results in Supplementary Tables S4/S5).

#### 3.2.3 Comparison of amygdala subnuclei volumes between-groups

Consistent with whole brain and total amygdalae volumes, subnuclei volumes were not normally distributed (*p*’s < .001). Regression models showed no significant differences between groups (Table 3).

**Table 3:** Linear regression models for analysis of subnuclei volumes (mm^3^) between-groups.

	**NEC** **(median (IQR))**	**PTSD** **(median (IQR))**	**β**	**95 % CI**	***p* value**
**Three Nuclei**					
Left Basolateral Nucleus	1393 (2 1 8)	1362 (2 0 8)	4.85	−14.8, 24.5	.63
Left Centromedial Nucleus	59 (16)	63 (14)	−2.0	−3.76, −0.34	.02*
Left Superficial Nucleus	249 (37)	244 (34)	0.34	−3.44, 4.13	.86
Right Basolateral Nucleus	1416 (2 1 2)	1398 (1 9 3)	5.52	−14.3, 25.3	.58
Right Centromedial Nucleus	68 (20)	68 (18)	.06	−1.74, 1.86	.95
Right Superficial Nucleus	257 (40)	253 (33)	.85	−3.16, 4.86	.68
**Nine Nuclei**					
Left Accessory Basal Nucleus	261 (37)	263 (38)	−1.27	−5.45, 2.91	0.55
Left Anterior Amygdaloid Area	50 (8)	48 (8)	0.77	−0.19, 1.72	0.12
Left Basal Nucleus	336 (1 7 6)	339 (1 6 8)	−0.36	−11.8, 11.1	0.95
Left Central Nucleus	42 (10)	44 (10)	−1.47	−2.63, −0.32	0.01*
Left Cortical Nucleus	23 (6)	24 (4)	−0.27	−0.80, 0.26	0.32
Left Cortico-amygdaloid Transition	178 (26)	171 (25)	−0.16	−3.03, 2.72	0.92
Left Lateral Nucleus	641 (1 0 8)	624 (1 0 0)	5.55	−4.52, 15.6	0.28
Left Medial Nucleus	17 (7)	18 (6)	−0.57	−1.31, 0.16	0.13
Left Paralaminar Nucleus	50 (8)	49 (9)	0.007	−0.82, 0.84	0.99
Right Accessory Basal Nucleus	271 (46)	272 (32)	0.63	−3.68, 4.95	0.77
Right Anterior Amygdaloid Area	54 (9)	52 (8)	0.73	−0.35, 1.80	0.19
Right Basal Nucleus	368 (1 7 2)	337 (1 6 8)	1.45	−9.99, 12.9	0.80
Right Central Nucleus	48 (12)	47 (9)	0.02	−1.17, 1.20	0.98
Right Cortical Nucleus	25 (7)	26 (4)	−0.19	−0.76, 0.38	0.51
Right Cortico-amygdaloid Transition	181 (29)	175 (22)	0.31	−2.68, 3.31	0.84
Right Lateral Nucleus	655 (94)	648 (98)	2.47	−7.58, 12.5	0.63
Right Medial Nucleus	19 (9)	20 (8)	0.04	−0.81, 0.90	0.92
Right Paralaminar Nucleus	49 (8)	49 (6)	0.16	−0.61, 0.93	0.68

*Note.* NEC = non-trauma-exposed controls. PTSD = posttraumatic stress disorder. IQR = interquartile range. CI = confidence interval. Linear regression was performed, with dependent variables adjusted for age, sex, and total brain volume. *p* value denotes difference in subnuclei volume between-groups. *Result significant at *p* < .05, but not after Bonferroni correction for multiple comparisons.

### 3.3 Analysis of nine nuclei

#### 3.3.1 Comparison of whole brain structural covariances between amygdala subnuclei and between-groups

In NEC, bilateral accessory-basal nuclei (vs. bilateral medial/left cortical nuclei) showed greater covariance with bilateral hippocampi and right amygdala (all *p*’s ≤ .0003, Table 4). In contrast, greater covariance in PTSD was found between bilateral accessory-basal nuclei (vs. right anterior-amygdaloid-.

**Table 4:** Significant differences in the covariance of brain regions with nine amygdala subnuclei in non-trauma-exposed controls.

**Amygdala Nucleus 1**	**Amygdala Nucleus 2**	**Brain Region**	***r*1**	***r* 2**	**z statistic**	***p* value**
Left Accessory-Basal	Left Medial	Left Hippocampus	.73	.31	4.66	.0001
		Right Hippocampus	.63	.10	5.26	.00001
	Left Cortical	Left Hippocampus	.73	.52	4.89	.00009
Right Accessory-Basal	Left Medial	Right Hippocampus	.71	.10	5.31	.000009
	Right Medial	Left Hippocampus	.70	.31	4.39	.0001
		Right Hippocampus	.71	.26	4.80	.0001
		Right Amygdala	.72	.32	4.40	.0001

Note. *r* = Pearson’s correlation. *r*1 = correlation between amygdala nucleus 1 and brain region. *r*2 = correlation between amygdala nucleus 2 and brain region. Z statistic is based on Dunn’s z. *p* value was FDR-corrected for the number of brain regions compared (1 0 5) and further Bonferroni-corrected for multiple subnuclei comparisons (significant *p***≤**.0003).

area, bilateral central/medial nuclei, right cortical, and left paralaminar nuclei) and bilateral amygdalae/right hippocampus; bilateral basal nuclei (vs. right anterior-amygdaloid-area, bilateral central/cortical/medial, and left paralaminar nuclei) and bilateral amygdalae/bilateral STG; left cortico-amygdaloid transition (vs. left anterior-amygdaloid-area and right medial nucleus) and bilateral amygdalae; and right paralaminar nucleus (vs. bilateral medial and left cortical nuclei) and bilateral STG/right MTG all (*p*’s ≤ .0003; complete results in Table 5). No between-group differences survived FDR-correction for multiple comparisons (uncorrected results in Supplementary Table S6).

**Table 5:** Significant differences in the covariance of brain regions with nine amygdala subnuclei in individuals with PTSD.

**Amygdala Nucleus 1**	**Amygdala Nucleus 2**	**Brain Region**	***r*1**	***r* 2**	**z statistic**	***p* value**
Left Accessory-Basal	Right AAA	Left Amygdala	.74	.18	5.00	.00005
	Left Central	Left Amygdala	.74	.39	4.83	.0001
		Right Hippocampus	.33	.02	4.93	.00007
	Right Central	Left Amygdala	.74	.19	4.94	.00007
	Right Cortical	Left Amygdala	.74	.22	5.05	.00004
		Right Amygdala	.69	.25	5.15	.00002
	Left Medial	Left Amygdala	.74	.28	4.44	.000005
		Right Amygdala	.69	.24	4.94	.00003
	Right Medial	Left Amygdala	.74	.14	5.38	.000007
		Right Amygdala	.69	.12	4.83	.00006
Right Accessory-Basal	Right Cortical	Right Amygdala	.69	.25	5.14	.00002
	Right Medial	Right Amygdala	.67	.12	4.73	.0002
		Right Hippocampus	.67	.14	4.52	.0003
	Left Paralaminar	Right Hippocampus	.67	.06	4.99	.00005
Left Basal	Right AAA	Left Amygdala	.74	.18	5.21	.00002
	Right Central	Left Amygdala	.74	.19	4.65	.0003
	Left Medial	Left Amygdala	.74	.28	4.64	.0002
		Left Superior Temporal Gyrus	.40	–.16	4.50	.0002
		Right Superior Temporal Gyrus	.39	–.16	4.47	.0002
	Right Medial	Left Amygdala	.74	.14	4.89	.00009
	Left Paralaminar	Left Amygdala	.74	.49	5.13	.00003
Right Basal	Right AAA	Right Amygdala	.82	.45	5.11	.00003
	Left Central	Right Amygdala	.82	.36	4.89	.00009
	Right Central	Right Amygdala	.82	.28	5.83	.0000005
	Left Cortical	Right Amygdala	.82	.43	4.50	.0003
		Left Superior Temporal Gyrus	.58	–.02	4.89	.00009
	Right Cortical	Right Amygdala	.82	.25	6.10	.00000009
	Left Medial	Right Amygdala	.82	.24	5.41	.000006
		Left Superior Temporal Gyrus	.58	–.16	5.18	.00001
	Right Medial	Right Amygdala	.82	.12	6.36	.00000002
		Left Superior Temporal Gyrus	.58	–.05	4.50	.0003
	Left Paralaminar	Right Amygdala	.82	.45	4.93	.00007
Left CAT	Left AAA	Left Amygdala	.69	.49	4.77	.0002
	Right Medial	Right Amygdala	.69	.12	4.62	.0003
Right Paralaminar	Left Medial	Left Superior Temporal Gyrus	.62	–.16	5.79	.0000006
		Right Superior Temporal Gyrus	.52	–.16	4.83	.00006
		Right Middle Temporal Gyrus	.47	–.17	4.44	.0003
	Right Medial	Left Superior Temporal Gyrus	.62	–.05	4.89	.00009
	Left Cortical	Left Superior Temporal Gyrus	.62	–.02	5.21	.00002

Note. *r* = Pearson’s correlation. *r*1 = correlation between amygdala nucleus 1 and brain region. *r*2 = correlation between amygdala nucleus 2 and brain region. Z statistic is based on Dunn’s z. *p* value was FDR-corrected for the number of brain regions compared (1 0 5) and further Bonferroni-corrected for multiple subnuclei comparisons (significant *p* ≤ .0003). AAA = Anterior-Amygdaloid Area. CAT = Cortico-Amygdaloid Transition.

#### 3.3.2 Comparison of amygdala subnuclei topological properties

Group differences for regional measures did not survive multiple comparison corrections (covariance matrices in Fig. 1; uncorrected results in Supplementary Table S7). Hub FDA showed higher nodal betweenness in PTSD for several areas, including the right basal nucleus (complete results in Supplementary Table S8). No subnuclei were found to be significant hubs in NEC.

#### 3.3.3 Comparison of amygdala subnuclei volumes between-groups

Consistent with above analyses, subnuclei volumes were not normally distributed (*p*’s < .001), and regression models showed no significant differences between-groups (Table 3).

## 4 Discussion

This study set out to investigate the structural covariance networks of amygdala subnuclei in PTSD vs. NEC. Contrary to our hypotheses, none of the group differences were found significant after multiple comparison correction; however, differences in covariance profiles between subnuclei within individual groups were found. When investigating subnuclei comparisons in NEC, differential covariance was found between the right BLA and left CMA for the right amygdala and between bilateral accessory-basal nuclei and bilateral medial/left cortical nuclei for bilateral hippocampi. In contrast, several differences were found in the PTSD group between various subnuclei and superior bilateral temporal and right frontal regions, and right middle and medial temporal regions. Topological analyses designated the right BLA and right basal nuclei as important hubs for nodal betweenness in PTSD but not in controls. Volumetric differences were not significant after correction for multiple comparisons. Together, these findings highlight unique amygdala subnuclei covariance patterns in both NEC and PTSD and underscore the importance of investigating the amygdala as a heterogenous structure to gain a more nuanced picture of the contribution of amygdala subnuclei to pathological brain functioning.

### 4.1 Whole brain structural covariances between amygdala subnuclei

When investigating three nuclei in NEC, differential covariance strengths were found between the BLA and CMA and the right amygdala. Comparatively, the BLA is the largest nucleus in rodents, with the lateral nucleus showing the most extensive intra-amygdaloid connections (Pitkänen et al., 1997)—an intra-network pattern that may be conserved in the amygdala in humans and result in greater covariance of the BLA with the entire amygdala. Covariance was also found to be greater between bilateral accessory-basal nuclei (vs. bilateral medial nuclei and left cortical nucleus) and bilateral hippocampi, and between the right accessory-basal nucleus (vs. right medial nucleus) and right amygdala. The basomedial nucleus (accessory-basal rodent homolog) has been associated with social stress/defeat (Ritger et al., 2023), the encoding of safe/aversive environmental contexts (Adhikari et al., 2015), and adaptive behavior to specific unpredictable situations (Ineichen et al., 2022). Furthermore, it has bidirectional projections with the hippocampus and innervates olfactory areas—similar to the cortical nucleus (Swanson and Petrovich, 1998, Pitkänen et al., 2000, Petrovich et al., 1996). In contrast, medial nuclei mediate innate emotional behavior through relaying olfactory information to reproductive and defensive hypothalamic nuclei (Keshavarzi et al., 2014). Higher covariance between the accessory-basal nucleus and amygdala/hippocampus in NEC may reflect the importance of this substrate for adaptive behavior in social/environmental contexts, over and above the behaviors mediated by medial/cortical nuclei and hippocampi/amygdala covariance.

Similar to NECs, greater covariance in PTSD was also found between the BLA/SFA (vs. CMA) and bilateral amygdalae in PTSD—possibly representing the lack of intra-amygdala connectivity of the CMA, given that central nuclei fibers are primarily efferent (Šimić et al., 2021). In PTSD, greater covariance was also found between the left BLA (vs. left CMA) and bilateral STG, right MTG, and right SFG. Meta-analyses investigating topological and volumetric properties of PTSD have found high nodal importance of the left STG (71) and low volumes for bilateral SFG (Bromis et al., 2018). Greater resting-state functional connectivity has also been reported between the BLA and STG in PTSD (Liu et al., 2021, Roy et al., 2015). Macaque MTG and STG homologs (TE and TA), largely innervate lateral subnuclei but do also project to basal nuclei (deCampo and Fudge, 2012). However, the right SFG does not project to the amygdala in rhesus monkeys (Porrino et al., 1981), possibly indicating that the relationship between the two structures is purely functional. When examining nine subnuclei, greater covariance was found between the bilateral basal/right paralaminar nuclei (vs. bilateral medial/left cortical nuclei) and bilateral STG, and between the right paralaminar nucleus (vs. left medial nucleus) and right MTG. Bilateral STG are particularly involved in multimodal sensory integration and echoic memory (Karnath, 2001, Inui et al., 2010): thus, covariance between basal nuclei and STG in PTSD might represent a biased substrate for auditory information over olfactory information that is processed through medial/cortical subnuclei (Swanson and Petrovich, 1998, Keshavarzi et al., 2014). This view finds support in the relative increase and decrease in size of temporal and olfactory processing areas, respectively, across phylogeny (Herzog and Van Hoesen, 1976). In contrast, the paralaminar nucleus has proven elusive, as it varies in position and prominence across species (deCampo and Fudge, 2012). Historically, it has been considered part of the basal nucleus and is thought to be involved in contextual learning (deCampo and Fudge, 2012), however, more research is required in primates and humans to further define both its functional and anatomical connectivity.

Additionally, greater covariance was found between the right SFA (vs. left CMA) and right hippocampus. In rodents, the three nuclei that comprise the SFA are innervated by the primary olfactory cortex (Müller and O'Rahilly, 2006, Cádiz-Moretti et al., 2016, Cádiz-Moretti et al., 2017). In PTSD (vs. TEC/NEC), individuals demonstrate higher distress to trauma specific odors (Cortese et al., 2015), suggesting that this SFA-hippocampal covariance may relate to encoding and access of traumatic memories associated with sensory cues. However, investigation of nine nuclei found greater covariance between bilateral accessory-basal nuclei and the right hippocampus (vs. left medial/central nuclei). In primates, the accessory-basal nucleus receives afferent projections from the hippocampus (Saunders et al., 1988); in humans, it is located beside the cortical nucleus and cortico-amygdaloid transition (Saygin et al., 2017) which are both part of the SFA. Their proximity may have resulted in an overlap of signal, or a dilution of signal when combining nine into three nuclei. Nonetheless, the differential covariance patterns found for nine nuclei build upon and clarify those seen for three nuclei and support similar functional relationships demonstrated in humans. Importantly, they highlight the need for ultra-high field MRI scanners in this examination, and the caution with which we need to interpret some animal findings.

Though within-group differences between subnuclei were found, no between-groups differences were found, contrary to fMRI studies with NEC (Brown et al., 2014, Koch et al., 2016, Leite et al., 2022, Liu et al., 2021, Neumeister et al., 2016, Rabellino et al., 2016, Nicholson et al., 2015, Nicholson et al., 2017, Nicholson et al., 2016). On reflection, this may be due to a few factors. It is well known that the nature of the control group moderates neural patterns in PTSD (Patel et al., 2012, Stark et al., 2015), and that the heterogeneity of trauma-type influences different symptom clusters (suggesting differing neuropathological profiles; (Henigsberg et al., 2001). Indeed, fMRI studies that that include trauma-exposed controls (unlike our study), do find more consistent amygdala activation in PTSD (Patel et al., 2012, Sripada et al., 2012, Brown et al., 2018, Shin et al., 2004, Bremner et al., 2005, Etkin and Wager, 2007). Additionally, although fMRI and structural covariance methods are analogous (both are based on statistical correlations in brain activity – the former, across time; the latter, across participants), morphological differences in amygdala subnuclei may not be as sensitive as functional differences, especially when probed by a task (Hayes et al., 2012). A further contributing factor may lie in our sample being underpowered as we do see differences at a less stringent threshold (supplementary results).

### 4.2 Topological properties of amygdala subnuclei

Graph analysis demonstrated that the influence of the right BLA and right basal nucleus in PTSD are greater than that of other amygdala subregions due to the higher number of connections that pass through them (Freeman, 1977). In rodents, it is well known that the basal nucleus is involved in encoding fear and reward and mediating anxiogenic effects (Šimić et al., 2021, Yang and Wang, 2017, Zhang et al., 2020). This, too, may underlie pathological processing in PTSD, particularly considering that the right basal nucleus has been found to be involved in the subjective experience of fear and to correlate with PTSD symptom severity (Frederikson and Furmark, 2003). Contrary to other studies, no significant group differences for global or regional network measures were found (Rakesh et al., 2021, Rakesh et al., 2023), which could be due to the stringent statistical threshold (Garamszegi, 2006) or sample sizes.

### 4.3 Volumetric comparisons of amygdala subnuclei between-groups

In line with our hypothesis, no group differences were found for amygdala subnuclei volumes. Evidence for volumetric differences has thus far been mixed, with a recent *meta*-analysis in a trauma-heterogenous sample finding a trend for smaller amygdalae volume in PTSD (with childhood trauma; 46). However, a recent study by Morey and colleagues (Morey et al., 2020) in 355 military veterans, found smaller bilateral lateral and paralaminar nuclei and larger bilateral central, cortical, and medial nuclei in veterans with PTSD. These results could suggest that volumetric differences of amygdala subnuclei may be moderated by trauma-type. Alternatively, they could also indicate the moderation of sex on amygdala volume, as the trend association in Logue et al’s (Logue et al., 2018) study was primarily driven by females, while Morey et al’s (Morey et al., 2020) study comprised 80 % males. Future studies may benefit from recruiting and stratifying samples based on sex and trauma-type to gauge the true effects of these factors on amygdala subnuclei volumes in PTSD. Additionally, previous work has found educational attainment to impact brain volume, however, we did not explicitly control for this in our analysis. This is because we controlled for total brain volume which is known to mediate the effect of education on regional brain size (Kim et al., 2023). Future work using larger cohorts should also analyze the impact of education on structural brain volume differences in PTSD.

### 4.4 Conclusion

This study highlights the importance of the accessory-basal, basal, and paralaminar nuclei to PTSD and illustrates the need to investigate the heterogeneity of the amygdala in the neuropathology of the disorder. While existing 3 T MRI protocols might be limited in their attempts to do this, future work could use ultra-high field fMRI to delineate differential functional roles of these structures for a more detailed understanding of amygdala subnuclei connectivity in PTSD.

## CRediT authorship contribution statement

**Elizabeth M. Haris:** Formal analysis, Visualization, Writing – original draft, Writing – review & editing. **Richard A. Bryant:** Supervision, Writing – review & editing. **Mayuresh S. Korgaonkar:** Conceptualization, Funding acquisition, Methodology, Supervision, Writing – review & editing.

## Data Availability

Data will be made available on request.
