# Supplementary material for: Structural covariance, topological organization, and volumetric features of amygdala subnuclei in posttraumatic stress disorder
Source: Neuroimage Clin. 2024 May 11;42:103619. doi: 10.1016/j.nicl.2024.103619 (PMC11108976; doi:10.1016/j.nicl.2024.103619)
Supplement: Supplementary Data 1 [file mmc1.docx]

**Supplemental Information**

**Methods and Materials**

**Table S1.** Gray matter volumes extracted with Freesurfer and used as regions of interest (ROI) for all analyses.

| Amygdala Areas | Cortical Areas | Subcortical Areas | |
| --- | --- | --- | --- |
| Accessory Basal Nucleus^*^ | L/R Frontal Pole | L/R Thalamus | |
| Anterior-Amygdaloid-Area^***^ | L/R Medial Orbitofrontal Cortex | L/R Ventral Diencephalon^1^ | |
| Basal Nucleus^*^ | L/R Lateral Orbitofrontal Cortex | L/R Hippocampus | |
| Central Nucleus^**^ | L/R Pars Orbitalis | L/R Parahippocampal Gyrus | |
| Cortical Nucleus^***^ | L/R Pars Opercularis | L/R Entorhinal Cortex | |
| Cortico-Amygdaloid Transition^***^ | L/R Pars Triangularis | L/R Amygdala | |
| Lateral Nucleus^*^ | L/R Middle Frontal Area (caudal) | L/R Accumbens | |
| Medial Nucleus^**^ | L/R Middle Frontal Area (rostral) | L/R Caudate | |
| Paralaminar Nucleus^*^ | L/R Superior Frontal Lobe | L/R Putamen | |
|  | L/R Precentral Gyrus | L/R Pallidum | |
|  | L/R Paracentral Gyrus | L/R Cerebellum | |
|  | L/R Anterior Cingulate Cortex (rostral) | Brain Stem | |
|  | L/R Anterior Cingulate Cortex (caudal) |  | |
|  | L/R Posterior Cingulate Cortex  L/R Isthmus Cingulate |  | |
|  | L/R Temporal Pole |  | |
|  | L/R Insula |  |  |
|  | L/R Inferior Temporal Lobe |  |  |
|  | L/R Middle Temporal Lobe |  |  |
|  | L/R Superior Temporal Lobe |  |  |
|  | L/R Bank Superior Temporal Sulcus |  |  |
|  | L/R Transverse Temporal Lobe |  |  |
|  | L/R Fusiform Gyrus |  |  |
|  | L/R Postcentral Gyrus |  |  |
|  | L/R Precuneus |  |  |
|  | L/R Cuneus |  |  |
|  | L/R Supramarginal Gyrus |  |  |
|  | L/R Inferior Parietal Cortex |  |  |
|  | L/R Superior Parietal Lobe |  |  |
|  | L/R Lingual Gyrus |  |  |
|  | L/R Pericalcarine Fissure |  |  |
|  | L/R Lateral Occipital Lobe |  | |

*Note.* L=left. R=right. ^*^Basolateral nucleus comprised of accessory-basal, basal, lateral, and paralaminar nuclei volumes. ^**^Centromedial nucleus comprised of central and medial nuclei volumes. ^***^Superficial nucleus comprised of anterior-amygdaloid-area, cortical nucleus, and cortico-amygdaloid transition volumes. ^1^Ventral diencephalon includes the following areas: hypothalamus, mammillary body, subthalamic nuclei, substantia nigra, red nucleus, lateral geniculate nucleus, and medial geniculate nucleus.

## Analysis of Three Nuclei

## *Comparison of amygdala subnuclei topological properties*

**Regional network topology.** Regional network measures calculated for nodal degree measure the number of connections of a node with the network, representing nodal importance. Those calculated for betweenness centrality measure the number of shortest paths that cross a node and represent nodes central for network communication, while those measuring local clustering represent nodes that tend to cluster together to create a smaller, complete graph. FDR-corrected *p*-values were used with a further Bonferroni correction to account for the three regional measures examined *(p*≤.02).

**Global network topology.** The graph-analysis toolbox (GAT; 56) was also used to compare global topological properties of networks using a structural covariance approach. Groups were compared on five non-normalized global network measures, including clustering coefficient (the degree to which nodes are interconnected, representing network segregation), characteristic path length (a quantification of the shortest path between two regions, representing network integration), small-worldness (the balance between network segregation and integration), and global and local efficiency (efficiency of information transfer across the whole brain and within smaller regions). FDR-corrected *p*-values were used with a further Bonferroni correction applied to account for the five global measures that were examined *(p*≤.01).

**Results**

**Analysis of Three Nuclei**

## *Comparison of whole brain structural covariances between amygdala subnuclei and between-groups*

Uncorrected comparisons between amygdala subnuclei between groups showed trends for lower structural covariance in PTSD (vs. healthy controls) of the left basolateral amygdala and bilateral hippocampi; higher structural covariance in PTSD between the right basolateral amygdala and temporal and parietal areas; lower covariance in PTSD between bilateral centromedial nuclei and bilateral hippocampi, bilateral cerebellum, frontal and anterior forebrain areas; and higher covariance in PTSD between the right superficial nucleus and frontal and parietal areas (Table S2).

## *Comparison of amygdala subnuclei topological properties*

**Regional network topology.** Uncorrected FDA of regional measures showed a trend of lower clustering and nodal degree in frontal and subcortical areas of the left hemisphere in PTSD, particularly for the left centromedial nucleus (*p*=.03; Table S3).

In addition to the significant hub analyses, uncorrected hub FDA also showed a pattern of higher betweenness centrality for frontal areas in the right hemisphere in PTSD (Table S4).

**Global network topology.** No corrected group differences were found for any global measures. Uncorrected results showed a trend towards higher network segregation measures across densities in those with PTSD (clustering coefficient, normalized clustering coefficient, local efficiency, small-worldness, and transitivity).

**Table S2.** Linear regression models showing the effect of education and total brain volume on subnuclei volumes (mm^3^) between-groups.

|  | **Education**  **(*p* value)** | **Brain Volume**  **(*p* value)** |
| --- | --- | --- |
| **Three Nuclei** |  |  |
| Left Basolateral Nucleus | .05^*^ | **<.001** |
| Left Centromedial Nucleus | .22 | **<.001** |
| Left Superficial Nucleus | .09 | **<.001** |
| Right Basolateral Nucleus | .20 | **<.001** |
| Right Centromedial Nucleus | .79 | **<.001** |
| Right Superficial Nucleus | .20 | **<.001** |
| **Nine Nuclei** |  | **<.001** |
| Left Accessory Basal Nucleus | .05^*^ | **<.001** |
| Left Anterior Amygdaloid Area | .28 | **<.001** |
| Left Basal Nucleus | .85 | **.002** |
| Left Central Nucleus | .22 | **<.001** |
| Left Cortical Nucleus | .18 | **<.001** |
| Left Cortico-amygdaloid Transition | .12 | **<.001** |
| Left Lateral Nucleus | .12 | **<.001** |
| Left Medial Nucleus | .50 | **<.001** |
| Left Paralaminar Nucleus | .12 | **<.001** |
| Right Accessory Basal Nucleus | .41 | **<.001** |
| Right Anterior Amygdaloid Area | .31 | **<.001** |
| Right Basal Nucleus | .95 | .01 |
| Right Central Nucleus | .24 | **<.001** |
| Right Cortical Nucleus | .58 | **<.001** |
| Right Cortico-amygdaloid Transition | .12 | **<.001** |
| Right Lateral Nucleus | .29 | **<.001** |
| Right Medial Nucleus | .82 | **<.001** |
| Right Paralaminar Nucleus | .25 | **<.001** |

*Note.* Supplementary linear regression performed to examine effect of education, with dependent variables adjusted for age, sex, education, and total brain volume. *p* value denotes the effect of education or total brain volume on subnuclei volume. *Result significant at *p*<.05, but not after Bonferroni correction for multiple comparisons (three nuclei: *p*≤.008; nine nuclei: *p*≤.003). Bold *p* values designate significant effects after multiple comparison correction.

**Table S3.** Uncorrected differences in the covariance of brain regions with three amygdala subnuclei in PTSD vs. healthy controls.

| Amygdala Nucleus 1 | Brain Region | *r*1 | *r*2 | z statistic | Uncorrected *p* value |
| --- | --- | --- | --- | --- | --- |
| Left Basolateral Nucleus | Left Hippocampus | .76 | .52 | 2.36 | .02 |
|  | Right Hippocampus | .61 | .25 | 2.55 | .01 |
|  | Left Caudate | –.18 | .18 | –2.06 | .04 |
| Right Basolateral Nucleus | Left Putamen | .21 | –.21 | 2.49 | .01 |
|  | Left Superior Temporal Gyrus | .21 | .56 | –2.38 | .02 |
|  | Right Middle Temporal Gyrus | .12 | .47 | –2.27 | .02 |
|  | Left Superior Parietal Area | –.14 | .22 | –2.14 | .03 |
|  | Right Inferior Temporal Gyrus | .10 | .41 | –1.96 | .05 |
| Left Centromedial Nucleus | Left Rostral Middle Frontal Gyrus | .19 | –.19 | 2.18 | .03 |
|  | Left Hippocampus | .55 | .26 | 2.02 | .04 |
|  | Right Hippocampus | .41 | .04 | 2.23 | .03 |
| Right Centromedial Nucleus | Left Cerebellum | .33 | –.09 | 2.49 | .01 |
|  | Right Cerebellum | .34 | –.10 | 2.60 | .009 |
|  | Left Ventral Diencephalon | .30 | –.04 | 2.04 | .04 |
| Left Superficial Nucleus | Right Parahippocampal Gyrus | .44 | .10 | 2.11 | .04 |
|  | Right Inferior Temporal Gyrus | .07 | .40 | –1.98 | .05 |
| Right Superficial Nucleus | Right Frontal Pole | –.30 | .07 | –2.14 | .03 |
|  | Left Superior Parietal Area | –.16 | .19 | –2.01 | .04 |

## Note. PTSD=posttraumatic stress disorder. *r*=Pearson’s correlation. *r*1=correlation between amygdala nucleus and brain region for healthy controls. *r*2=correlation between amygdala nucleus and brain region for PTSD. Z/*p* statistics based on Fisher’s z.

**Table S4.** Normalized and uncorrected whole brain regional measures (for three amygdala subnuclei), representing clustering, nodal degree, and betweenness centrality of network nodes in PTSD vs. healthy controls (HC).

| Clustering Coefficient | Nodal Degree | Betweenness Centrality | Brain Region | Results |
| --- | --- | --- | --- | --- |
| 0.032 |  |  | L Centromedial Nucleus | PTSD < HC |
| 0.007 | 0.017 | 0.048 | R Insula | PTSD > HC, PTSD < HC, PTSD < HC |
| 0.052 |  |  | L Orbitofrontal Cortex (lateral) | PTSD > HC |
| 0.045 |  |  | R Orbitofrontal Cortex (lateral) | PTSD > HC |
| 0.035 |  |  | L Anterior Cingulate Cortex (caudal) | PTSD < HC |
| 0.053 | 0.003 | 0.030 | R Posterior Cingulate Cortex | PTSD < HC, PTSD < HC, PTSD > HC |
| 0.017 |  |  | L Amygdala | PTSD < HC |
| 0.029 |  |  | R Caudate | PTSD > HC |
|  | 0.053 |  | L Frontal Pole | PTSD > HC |
|  | 0.039 |  | L Middle Frontal Gyrus | PTSD < HC |
|  | 0.044 |  | R Middle Frontal Gyrus | PTSD < HC |
|  | 0.004 |  | R Middle Frontal Gyrus (rostral) | PTSD > HC |
|  | 0.034 |  | L Anterior Cingulate Cortex (rostral) | PTSD < HC |
|  | 0.006 | 0.018 | L Posterior Cingulate Cortex | PTSD < HC, PTSD < HC |
|  | 0.030 |  | R Middle Temporal Gyrus | PTSD > HC |
|  | 0.041 |  | L Nucleus Accumbens | PTSD < HC |
|  | 0.044 |  | Brain Stem | PTSD < HC |
|  |  | 0.005 | L Paracentral Lobule | PTSD > HC |
|  |  | 0.018 | L Parahippocampal Gyrus | PTSD > HC |
|  |  | 0.043 | L Cerebellum | PTSD < HC |

*Note:* Values represent uncorrected *p*<0.05. Clustering signifies the embeddedness of a node in the network (and is based upon the proportion of connections among a node’s neighbors). Nodal degree represents the density of connections of a node. Betweenness signifies the centrality of the node to network communication (based upon the number of shortest paths that traverse it).

**Table S5.** Significant hubs for both PTSD and healthy controls for three amygdala subnuclei. Hubs are areas of the brain crucial to the communication of the network, specifically, nodes that act as ‘bridges’ for efficient communication (betweenness centrality), and nodes that are highly connected within the network (nodal degree).

| **Betweenness HC** | **Degree HC** | **Betweenness PTSD** | **Degree PTSD** |
| --- | --- | --- | --- |
| L Middle Frontal Gyrus (rostral) | L Middle Frontal Gyrus (rostral) | R Basolateral Nucleus | L Superior Frontal Gyrus |
| R Thalamus |  | L Superior Frontal Gyrus | R Middle Frontal Gyrus (rostral) |
| L Nucleus Accumbens |  | R Inferior Temporal Gyrus |  |
|  |  | R Middle Frontal Gyrus (rostral) |  |
|  |  | R Superior Frontal Gyrus |  |

*Note.* Hubs are corrected using functional data analysis.

**Analysis of Nine Nuclei**

## *Comparison of whole brain structural covariances between amygdala subnuclei and between-groups*

Uncorrected comparisons between amygdala subnuclei between groups showed trends for higher covariance in PTSD of inferior and anterior amygdala subnuclei with temporal gyri, superior frontal, and superior parietal areas, and lower structural covariance in PTSD between superior and posterior amygdala subnuclei with hippocampi, cerebellum, middle frontal, and midline frontal and parietal structures (complete results in Table S5).

## *Comparison of amygdala subnuclei topological properties*

## Regional network topology. Uncorrected FDA additionally showed a trend for individuals with PTSD (vs. controls) to have lower normalized clustering coefficient for the right basal, central, and cortical amygdala nuclei, and right inferior frontal gyrus. Those with PTSD also showed a trend towards greater nodal betweenness for the right accessory-basal and basal amygdala nuclei, greater nodal degree of bilateral middle temporal gyri, and lower nodal degree of bilateral posterior cingulate cortex. Regional results and hub analysis results can be found in Tables S6 and S7, respectively.

**Global network topology.** No corrected group differences were found for any global measures. Uncorrected results again showed a trend towards higher network segregation measures in those with PTSD (clustering coefficient, normalized clustering coefficient, local efficiency, small-worldness, and transitivity), but only at lower densities.

**Table S6.** Uncorrected differences in the covariance of brain regions with nine amygdala subnuclei in PTSD vs. healthy controls.

| Amygdala Nucleus 1 | Brain Region | *r*1 | *r*2 | z statistic | Uncorrected *p* value |
| --- | --- | --- | --- | --- | --- |
| Left Anterior-Amygdaloid-Area | Left Superior Temporal Gyrus | .04 | .37 | –1.97 | .05 |
| Right Anterior-Amygdaloid-Area | Left Superior Parietal Area | –.17 | .20 | –2.15 | .03 |
| Left Accessory-Basal Nucleus | Right Hippocampus | .63 | .33 | 2.26 | .02 |
|  | Left Amygdala | .55 | .74 | –1.96 | .05 |
| Right Accessory-Basal Nucleus | Left Temporal Pole | –.13 | .21 | –1.98 | .05 |
| Left Basal Nucleus | Left Hippocampus | .61 | .22 | 2.80 | .005 |
|  | Right Hippocampus | .73 | .51 | 2.03 | .04 |
|  | Left Caudate | –.14 | .21 | –2.01 | .05 |
| Right Basal Nucleus | Left Superior Temporal Gyrus | .22 | .58 | –2.53 | .01 |
|  | Left Superior Parietal Area | –.20 | .22 | –2.43 | .02 |
|  | Right Inferior Temporal Gyrus | .04 | .42 | –2.34 | .02 |
|  | Left Putamen | .18 | –.20 | 2.23 | .03 |
|  | Left Middle Temporal Gyrus | .13 | .44 | –1.99 | .05 |
|  | Right Middle Temporal Gyrus | .14 | .47 | –2.13 | .03 |
|  | Left Superior Frontal Gyrus | .01 | .35 | –2.04 | .04 |
| Left Corticoamygdaloid Transition | Left Hippocampus | .65 | .38 | 2.14 | .03 |
|  | Right Rostral Middle Frontal Gyrus | –.09 | .37 | –2.14 | .03 |
|  | Right Frontal Pole | –.33 | .03 | –2.13 | .03 |
| Left Central Nucleus | Left Hippocampus | .59 | .02 | 2.37 | .02 |
|  | Right Hippocampus | .52 | .26 | 3.15 | .002 |
|  | Right Fusiform Gyrus | –.01 | .31 | –1.92 | .05 |
| Right Central Nucleus | Left Cerebellum | .36 | –.03 | 2.30 | .02 |
|  | Right Cerebellum | .36 | –.08 | 2.60 | .009 |
|  | Right Amygdala | .57 | .28 | 2.05 | .04 |
|  | Left Lingual Gyrus | .14 | –.20 | 1.97 | .05 |
| Right Cortical Nucleus | Left Ventral Diencephalon | .32 | .18 | 2.95 | .003 |
|  | Right Superior Parietal Area | .15 | .23 | 2.20 | .03 |
|  | Right Precuneus | .10 | .24 | 1.96 | .05 |
| Left Lateral Nucleus | Right Cerebellum | .006 | .34 | –1.97 | .05 |
|  | Right Middle Temporal Gyrus | .16 | .46 | –1.95 | .05 |
| Right Lateral Nucleus | Left Putamen | .24 | –.23 | 2.76 | .006 |
|  | Left Posterior Cingulate | .26 | –.12 | 2.21 | .03 |
|  | Right Middle Temporal Gyrus | .09 | .41 | –1.97 | .05 |
| Left Medial Nucleus | Left Rostral Middle Frontal Gyrus | .15 | –.24 | 2.26 | .02 |
|  | Left Frontal Pole | .06 | –.28 | 2.02 | .04 |
|  | Left Rostral Anterior Cingulate | –.28 | .05 | –1.96 | .05 |
| Right Medial Nucleus | Left Ventral Diencephalon | .17 | –.21 | 2.17 | .03 |
|  | Left Cerebellum | .20 | –.16 | 2.20 | .03 |
| Left Paralaminar Nucleus | Right Hippocampus | .38 | .06 | 1.94 | .05 |
| Right Paralaminar Nucleus | Left Superior Temporal Gyrus | .21 | .62 | –2.94 | .003 |
|  | Left Middle Temporal Gyrus | .09 | .52 | –2.77 | .006 |
|  | Right Inferior Temporal Gyrus | .09 | .50 | –2.60 | .009 |
|  | Left Superior Parietal Area | –.26 | .11 | –2.18 | .03 |
|  | Left Putamen | .19 | –.17 | 2.11 | .04 |
|  | Right Rostral Middle Frontal Gyrus | .07 | .38 | –1.93 | .05 |

## Note. PTSD=posttraumatic stress disorder. *r*=Pearson’s correlation. *r*1=correlation between amygdala nucleus and brain region for healthy controls. *r*2=correlation between amygdala nucleus and brain region for PTSD. Z/*p* statistics based on Fisher’s z. Ventral diencephalon encompasses several areas, including, hypothalamus, basal forebrain, sublenticular extended amygdala, and ventral tegmentum.

**Table S7.** Normalized and uncorrected whole brain regional measures (for three amygdala subnuclei), representing clustering, nodal degree, and betweenness centrality of network nodes in PTSD vs. healthy controls (HC).

| Clustering Coefficient | Nodal Degree | Betweenness Centrality | Brain Region | Results |
| --- | --- | --- | --- | --- |
| 0.013 |  | 0.001 | R Basal Nucleus | PTSD < HC, PTSD > HC |
| 0.043 |  |  | R Central Nucleus | PTSD < HC |
| 0.022 |  |  | R Cortical Nucleus | PTSD < HC |
| 0.051 |  |  | L Middle Frontal Gyrus (rostral) | PTSD > HC |
| 0.021 |  |  | R Pars Triangularis | PTSD > HC |
| 0.007 |  |  | R Insula | PTSD > HC |
| 0.017 |  |  | L Orbitofrontal Cortex (lateral) | PTSD > HC |
| 0.035 |  |  | L Anterior Cingulate Cortex (caudal) | PTSD < HC |
| 0.007 |  |  | L Amygdala | PTSD < HC |
|  | 0.002 |  | R Middle Frontal Gyrus (rostral) | PTSD > HC |
|  | 0.017 |  | L Posterior Cingulate Cortex | PTSD < HC |
|  | 0.003 |  | R Posterior Cingulate Cortex | PTSD < HC |
|  | 0.028 |  | L Middle Temporal Gyrus | PTSD > HC |
|  | 0.017 |  | R Middle Temporal Gyrus | PTSD > HC |
|  | 0.024 |  | L Nucleus Accumbens | PTSD < HC |
|  |  | 0.041 | R Accessory Basal Nucleus | PTSD > HC |

*Note:* Values represent uncorrected *p*<0.05. Clustering signifies the embeddedness of a node in the network (and is based upon the proportion of connections among a node’s neighbors). Nodal degree represents the density of connections of a node. Betweenness signifies the centrality of the node to network communication (based upon the number of shortest paths that traverse it).

**Table S8.** Significant hubs for both PTSD and healthy controls for nine amygdala subnuclei. Hubs are areas of the brain crucial to the communication of the network, specifically, nodes that act as ‘bridges’ for efficient communication (betweenness centrality), and nodes that are highly connected within the network (nodal degree).

| **Betweenness HC** | **Betweenness PTSD** | **Degree PTSD** |
| --- | --- | --- |
| L Middle Frontal Gyrus (rostral) | R Basal Nucleus | L Superior Frontal Gyrus |
| L Hippocampus | L Superior Frontal Gyrus | R Inferior Temporal Gyrus |
| L Nucleus Accumbens | R Inferior Temporal Gyrus |  |
| L Ventral Diencephalon | R Superior Frontal Gyrus |  |
| R Hippocampus |  |  |

*Note.* Hubs are corrected using functional data analysis.
